# From Deficiency to Therapy: Systemic Consequences of ALAS1 Disruption and the Protective Role of 5-ALA

**DOI:** 10.3390/life15081259

**Published:** 2025-08-07

**Authors:** Koen van Wijk, Osamu Nakajima

**Affiliations:** Research Center for Molecular Genetics, Institute for Promotion of Medical Science Research, Faculty of Medicine, Yamagata University, Yamagata 990-9585, Japan; mxm189@med.id.yamagata-u.ac.jp

**Keywords:** 5-aminolevulinic acid, mitochondria, skeletal muscle, autophagy, insulin resistance, glucose intolerance, native immunity, multi-organ, aging, sarcopenia

## Abstract

Heme, an essential prosthetic group involved in mitochondrial respiration and transcriptional regulation, is synthesized via the rate-limiting enzyme 5-aminolevulinic acid synthase (ALAS). Utilizing heterozygous mouse models for ALAS1 and ALAS2, our studies have revealed diverse systemic consequences of chronic heme deficiency. ALAS1-heterozygous (ALAS1+/−) mice develop metabolic dysfunction characterized by insulin resistance, glucose intolerance, and abnormal glycogen accumulation, linked mechanistically to reduced AMP-activated protein kinase (AMPK) signaling. These mice also exhibit pronounced mitochondrial dysfunction, impaired autophagy, and accelerated aging phenotypes, including sarcopenia and metabolic decline, highlighting heme’s role as a critical metabolic regulator. Additionally, ALAS2 heterozygosity (ALAS2+/−) leads to impaired erythropoiesis, resulting in anemia and ineffective iron utilization. Importantly, supplementation with the heme precursor 5-aminolevulinic acid (5-ALA) significantly mitigates ALAS1+/− phenotypes, restoring metabolic function, mitochondrial health, autophagy, and immune competence. This review encapsulates key findings from our group’s research together with advances made by multiple research groups over the past decade, collectively establishing heme homeostasis as a central regulator of systemic physiology and highlighting the therapeutic potential of 5-ALA in treating heme-deficient pathologies.

## 1. Heme Biosynthesis and Central Role in Cellular Metabolism

Heme is an essential iron-containing prosthetic group that plays crucial roles in diverse biological processes. It forms the prosthetic group of hemoglobin and myoglobin (oxygen transport/storage) and is a cofactor for numerous enzymes, including complex II, III, and IV of the mitochondrial electron transport chain (ETC) and detoxification enzymes like cytochromes P450, which help with xenobiotic detoxification and steroid metabolism [1,2].

Heme facilitates electron transfer and redox reactions necessary for cellular respiration and energy production [3,4,5]. In addition to these roles, heme also serves as a signaling molecule that can bind multiple transcription factors, for example, the heme-responsive repressor Bach1, to regulate gene expression [6,7,8].

Heme is synthesized via an eight-step pathway spanning the mitochondria and cytosol. The first and rate-limiting step is the condensation of glycine and succinyl-CoA to form 5-aminolevulinic acid (5-ALA), catalyzed by ALA synthase (ALAS) in the mitochondrial matrix. Vertebrates have two ALAS isoforms: ALAS1 is ubiquitously expressed and subject to feedback inhibition by heme [9], whereas ALAS2 is expressed predominantly in erythroid cells to support massive hemoglobin production during erythropoiesis [10,11]. The subsequent steps produce various porphyrin intermediates in mitochondria and cytosol, culminating in the insertion of ferrous iron into protoporphyrin IX by ferrochelatase in mitochondria to yield heme [12]. This multi-step pathway is coordinated with iron availability and globin synthesis in erythroid cells. Disruption at any step can lead to the accumulation of toxic precursors or iron, and deficiencies in heme synthesis underlie disorders such as sideroblastic anemia and porphyria [13]. Thus, heme homeostasis is tightly regulated to meet metabolic demands while preventing free heme toxicity [14].

Beyond its role as the first committed intermediate in heme biosynthesis, 5-ALA is gaining recognition as a therapeutic agent with potential in metabolic, inflammatory, and mitochondrial disease (Kuryata et al. 2024) [15]. This review focuses on how endogenous heme deficiency, mainly through studies involving ALAS1 disruption, leads to systemic dysfunction. Though discussed to a lesser extent, findings from ALAS2-related disorders also offer insights into the consequences of impaired heme biosynthesis. We additionally explore how 5-ALA supplementation may help reverse or alleviate these effects.

## 2. Essential Role of ALAS1 and ALAS2 Isozymes in Embryogenesis and Development

The indispensable nature of heme is highlighted by the phenotypes of ALAS-knockout mice. *Alas1* knockout mice (ALAS1−/−) die by embryonic day 7.5 with developmental arrest [16]. The early lethality of ALAS1-null embryos demonstrates that the ubiquitous ALAS1 is essential for producing basal levels of 5-ALA/heme in most tissues, a function that cannot be compensated by the erythroid-specific ALAS2. Heterozygous ALAS1+/− mice, by contrast, are viable and reach adulthood but exhibit only ~50% of normal ALAS1 mRNA in skeletal muscle, liver, and adipose tissues. These ALAS1+/− mice serve as a model of systemic heme deficiency in adult life [17,18]. Knockout of the erythroid-specific *Alas2* gene similarly leads to embryonic lethality. Male mice lacking ALAS2, which is linked to the X-chromosome, die by embryonic day ~11.5 due to severe anemia [10]. Without ALAS2, yolk sac erythroblasts cannot synthesize hemoglobin, leading to fatal anemia in mid-gestation. Nakajima et al. reported that ALAS2-null embryos have dramatically reduced red blood cell production and accumulate excess iron in the cytosol of erythroid precursors [10]. It is noteworthy that, in humans, a common ALAS2 deficiency, X-linked sideroblastic anemia (XLSA), has ring sideroblasts, erythroblasts accumulating iron in mitochondria, due to impaired heme production [19]. Nakajima et al. showed that ring sideroblasts and siderocytes were found in embryos of partially transgenically rescued ALAS2-deficient mice [20].

In vitro differentiation of ALAS2-deficient embryonic stem cells confirmed an increase in non-heme (free) iron and heightened oxidative stress in erythroblasts with reduced heme, with some impairment to terminal erythroid maturation after enucleation [21]. These findings illustrate that heme synthesis is absolutely required for effective erythropoiesis—its absence causes iron mismanagement and oxidative damage, culminating in embryonic lethality. Together, the ALAS knockout models establish that heme is indispensable for life, and each ALAS isozyme performs a non-redundant, tissue-specific role in supplying heme [22].

## 3. Systemic Metabolic Dysfunction Caused by Heme Deficiency

One of the major insights from ALAS1+/− mice is that heme deficiency can lead to metabolic disease phenotypes in adulthood. Despite showing no phenotypical changes when young, ALAS1+/− mice older than ~20–25 weeks develop impaired glucose tolerance and insulin resistance without obesity. This phenotype resembles pre-type 2 diabetes in terms of elevated blood glucose and blunted insulin responsiveness but notably occurs in lean mice. Oral supplementation with 5-ALA, which boosts heme synthesis, can temporarily normalize glucose tolerance and insulin sensitivity in our ALAS1+/− mice, confirming that the metabolic defect is indeed due to heme insufficiency. Below we detail how metabolic pathways are affected by heme deficiency [17].

### 3.1. Insulin Resistance and Glucose Intolerance in ALAS1+/− Mice

ALAS1+/− “heme-deficient” mice in mid-life show an impaired ability to dispose of glucose in tolerance tests. Insulin resistance is increased, as indicated by insulin tolerance testing, while insulin levels during glucose tolerance testing remain unaffected. Unlike classical type 2 diabetes models, these mice do not develop obesity; in fact, some weight loss occurs after 75 weeks of age (see sarcopenia section) [23]. The metabolic disturbances can be rescued by restoring heme precursor levels (via 5-ALA), linking them causally to heme depletion. Beyond ALAS1 deficiency, similar metabolic phenotypes have been observed in mice lacking progesterone receptor membrane component 2 (PGRMC2), a heme chaperone and trafficking protein. PGRMC2 knockout impairs labile heme distribution, disrupts mitochondrial respiration in adipocytes, and reduces thermogenic gene expression, outcomes that closely mirror the energy metabolism deficits seen in ALAS1+/− mice. These findings support the broader concept that intracellular heme handling is critical for systemic metabolic regulation [24]. Mechanistically, heme deficiency in muscle appears to blunt insulin signaling. Muscle is the primary tissue for insulin-stimulated glucose uptake, and in aged ALAS1+/− mice, downstream effectors of the insulin pathway (Akt and GSK3β) were less activated during a basal state but not during insulin tolerance testing. This suggests an impairment in tonic (basal) signaling, which may involve altered redox status or transcriptional changes due to low heme [17]. Interestingly, a 2014 study by Ju et al. showed that hemin was able to increase insulin sensitivity in skeletal muscle through heme oxygenase-1 (HO-1) induction and mitigate some of the negative effects from a high-fat diet, which was reflected in the amounts of triglycerides and oxidative stress markers [25]. Indeed, Fujii et al., in 2017, similarly found that 5-ALA supplementation in mice helped treat glucose intolerance as the mitochondria were activated, increasing oxidative phosphorylation, muscle performance, and muscle size [26], thus confirming that inadequate heme may impair insulin action by intrinsic defects in muscle energy metabolism [17]. In line with these findings, we also observed alterations in glycogen metabolism, a key determinant of muscle glucose handling, suggesting that heme deficiency may disrupt not only insulin signaling but also downstream glucose storage and utilization.

### 3.2. Glycogen Storage Dysregulation via AMPK Suppression in Heme-Deficient Muscle

In addition to disrupted glucose handling and insulin signaling, we observed aberrant glycogen accumulation in skeletal muscle of heme-deficient ALAS1+/− mice. Despite normal insulin signaling, aged heme-deficient muscle shows excessive glycogen deposits, implying a block in glycogen utilization or an overactive glycogen synthesis pathway [27]. One possible explanation could be elevated glycogen synthase (GYS1) activity due to reduced phosphorylation (inactivation) by AMPK. Normally, AMPK activation, during exercise or energy stress, phosphorylates and inhibits GYS1, limiting glycogen synthesis when energy is needed. In heme-deficient muscle, chronic AMPK suppression [23] leads to elevated unphosphorylated GYS1, driving pathological glycogen storage. This excess intramuscular glycogen may interfere with insulin-mediated glucose uptake [28]. Supporting this mechanism, treating heme-deficient mice with AICAR (AMPK activator) mimics the effects on GYS1/glycogen and insulin responsiveness. Pharmacological AMPK activation alleviated insulin resistance in vitro, reinforcing the conclusion that disrupted glycogen handling, due to low AMPK signaling, contributes to the glucose intolerance in vivo [17]. Thus, heme deficiency deranges muscle fuel management by disabling an important energy-sensing pathway (AMPK) and skewing the balance between glucose storage and oxidation.

## 4. Heme Deficiency-Induced Mitochondrial Defects in Skeletal Muscle

Heme deficiency profoundly affects mitochondrial structure and function, particularly in metabolically active, mitochondria-rich tissues such as muscle [17]. As stated before, heme is required for the assembly of several proteins that make up the ETC [17,29]. In a study by Atamna et al., IMR90 cells (human fibroblast cells) were used to investigate the effects of heme deficiency on complex IV and other hemoprotein assembly. Treating the IMR90 cells with NMP, a ferrochelatase inhibitor, prevented proper assembly of complex IV and led to compromised respiration, thus showing the importance of incorporating heme in the oxidative phosphorylation process. Moreover, Atamna et al. also showed that this process was more affected in older cells than in younger cells, because the rate of protein assembly was more affected in old cells; this aligns with our findings that heme deficiency affects other processes with aging [30]. In skeletal muscle of aged ALAS1+/− mice, the structure of the mitochondria was found to be abnormal; they appeared atrophied, with indistinct cristae, and had a markedly reduced electron transport capacity. Mitochondrial DNA copy number and expression of cytochrome c oxidase subunits were significantly decreased in heme-deficient muscle [17]. Functionally, heme-deficient mice exhibit lower exercise endurance, likely as a result of impaired muscle oxidative phosphorylation [17,23]. 5-ALA has been shown to partially reverse these mitochondrial deficits and improve skeletal muscle function [26].

At the molecular level, heme deficiency activates a mitochondrial stress response. When ETCcomplexes lack their heme prosthetic groups, electrons can leak and generate reactive oxygen species (ROS). Free iron, unbound due to failed heme incorporation, may catalyze Fenton chemistry [31], compounding oxidative stress. Chronic ROS accumulation impairs mitochondrial biogenesis and can destabilize mitochondrial DNA [32]. Models of both heme overload and deficiency in muscle show that disturbed heme homeostasis disrupts mitochondrial function and muscle fiber composition. In mice with skeletal muscle-specific deletion of HO-1 (MHO-1), heme accumulation, impaired ETC complex II expression, and enlarged dysfunctional mitochondria were observed. These mice also show a shift toward glycolytic muscle fibers (less oxidative) and reduced satellite cell signaling [33]. In contrast, deletion of a key heme exporter, feline leukemia virus subgroup C receptor 1a (Flvcr1a), in mice resulted in lower ALAS1 activity, reduced cytosolic heme, and impaired complex II and IV activity. Aged Flvcr1a KO mice also exhibit reduced grip strength and a shift towards oxidative muscle fiber types (less glycolytic), which is similar to changes seen with aging in mice [34,35]. Interestingly, both MHO-1 and Flvcr1a models develop mitochondrial dysfunction, despite opposite directions of heme imbalance.

These mechanisms remain incompletely understood but may involve changes in mitochondrial morphology, autophagy, and myoglobin content. These findings suggest that disrupted heme homeostasis can impair mitochondrial respiration and shift muscle phenotype, supporting some of our observations in the ALAS1+/− model. In summary, disrupted heme balance, whether by shortage or abundance, compromises mitochondrial function in muscle, leading to morphological degeneration and reduced aerobic capacity. Given the central role of mitochondria in muscle metabolism, their dysfunction necessitates robust quality control mechanisms, particularly mitochondrial autophagy (mitophagy), to maintain cellular homeostasis. We next address how heme deficiency impacts these essential clearance pathways, further compounding muscle pathology [36].

## 5. Suppressed Autophagy in Heme Deficiency: The Role of AMPK Signaling

Heme deficiency also interferes with cellular quality control mechanisms such as autophagy. Autophagy, including the selective removal of mitochondria called mitophagy, when altered, can lead to muscle diseases characterized by abnormal mitochondrial accumulation and vacuole formation, which contributes to the degeneration and weakening of myofibers and progressively worsens in aging muscles [37,38]. Autophagy is crucial in muscle to recycle damaged organelles and proteins, especially during fasting or exercise. In aged ALAS1+/− muscle, autophagy was found to be blunted: levels of LC3-II, a well-known autophagosome marker, fail to appropriately increase under fasting conditions, indicating reduced autophagosome formation. Consistently, ALAS1 knockdown in C2C12 muscle cells, or treatment of cells with succinyl acetone (SA), a heme synthesis inhibitor, also led to lower LC3 protein expression. Importantly, 5-ALA supplementation was able to restore LC3 protein expression in ALAS1+/− mice, linking the autophagy defect directly to heme availability [23].

Autophagy impairment is mechanistically tied to AMPK, as it is the main initiator of the autophagic process. Like stated in Section 3 in regard to glycogen, in ALAS1+/− muscle, the active form of AMPK (pAMPK) is significantly decreased [23]. AMPK activation promotes autophagy by phosphorylating ULK1, Beclin1, and components of the VPS34 complex, thereby initiating autophagy. Thus, low AMPK activity provides a plausible cause for suppressed autophagy [39]. Supporting this, treatment of ALAS1-deficient C2C12 muscle cells with AICAR restored LC3 levels and autophagy, whereas dorsomorphin (an AMPK inhibitor) reduced LC3 in control cells [23]. The data indicate that heme deficiency leads to AMPK inactivation, likely due to altered AMP/ATP balance or redox signals, and this in turn hampers autophagic clearance of dysfunctional mitochondria [30]. The accumulation of abnormal, non-functional mitochondria and proteins could exacerbate muscle insulin resistance and atrophy.

Overall, heme deficiency appears to accelerate a vicious cycle of energy stress: mitochondrial dysfunction in our mice coincides with increased ATP levels and inactivation of AMPK, leading to poor autophagy and further mitochondrial damage. This remains a paradox, as reduced heme levels (and presumably less heme incorporation into ETC complexes) would typically impair ATP production. One possible explanation comes from Graber et al., who showed that deletion of DEP domain-containing 5 (DEPC5) protein, a negative regulator of mammalian target of rapamycin complex 1 (mTORC1), led to constitutively active mTORC1. This resulted in increased mitochondrial respiratory capacity, even though muscle quality was reduced, possibly due to decreased autophagy and accumulation of damaged organelles [40]. A similar mechanism might underlie the ATP increase in ALAS1+/− mice, although our mice display reduced, not hypertrophied, muscle mass and mitochondrial disfunction, suggesting other mechanisms must be at play.

Another hypothesis involves the heme-regulated inhibitor kinase (HRI), which normally functions in erythroid cells but has also been implicated in the mitochondrial stress response. In conditions of heme deficiency, HRI may become active, triggering the integrated stress response (ISR) and suppressing global protein synthesis [41]. This could reduce ATP consumption, resulting in the observed ATP surplus in ALAS1+/− mice. Finally, since heme is known to activate AMPK via the ATF4/HO-1 axis, it remains unclear why AMPK remains inactive in our model [42]. Whether insufficient heme fails to engage this pathway or whether other regulatory brakes exist still needs to be investigated. A more comprehensive understanding of how heme status intersects with mTORC1, HRI, and AMPK signaling will be essential to resolve this apparent contradiction and clarify the mechanisms driving energy imbalance in heme-deficient muscle.

## 6. Heme Deficiency as a Driver of Age-Associated Decline in Muscle Function and Metabolism

Sarcopenia, the age-related loss of skeletal muscle mass and strength, is exacerbated by heme deficiency. ALAS1+/− mice, which produce less heme due to reduced ALAS1 expression, exhibit considerable declines in muscle mass and grip strength by 18 months of age, compared to wild-type controls. Importantly, these phenotypes are not present in younger ALAS1+/− mice, suggesting that aging and heme insufficiency act synergistically. This raises the possibility that dietary 5-ALA or heme precursor supplementation in older adults might help preserve muscle mass and function as an “anti-aging” effect was observed in mouse models that warrants further investigation [23].

Even in normal wild-type mice, aging is associated with changes in heme metabolism, hepatic ALAS1 activity, and expression decline in older rodents [22], and ALAS1 mRNA and free heme levels drop in skeletal muscle with age [18]. ALAS1+/− mice essentially start life at a heme disadvantage and therefore reach a critical threshold of heme deficiency earlier in life than wild-type mice. Interestingly, the above-mentioned Flvcr1a-deficient mice similarly showed an age-related decline in muscle strength, where Mistretta et al. compared mice at 2 months and 10 months of age, again showing a parallel with our studies related to heme’s role in aging [34].

These aging phenotypes, sarcopenia, insulin resistance, and mitochondrial dysfunction, parallel those of our ALAS1+/− mice described in Section 3 and Section 4 and may reflect cumulative consequences of heme insufficiency over time with aging.

Supplementation with 5-ALA not only corrected these age-related phenotypes but also restored mitochondrial morphology and oxidative function (see Section 4). For instance, Fujii et al. employed prolonged 5-ALA treatment, which increased muscle mass and improved glucose tolerance in wild-type mice aged 10–15 weeks [26], suggesting that even physiologic heme levels in adulthood might be suboptimal for peak tissue function. This concept is further supported by parallels in humans: elderly individuals frequently exhibit anemia of unknown cause [43], which may reflect impaired heme production, immune senescence, and sarcopenia [44]. It is conceivable that age-related decline in ALAS1 or other heme biosynthesis components contributes to these syndromes. If so, interventions that boost heme synthesis, such as 5-ALA supplementation, may provide a strategy to improve health span and lifespan. In summary, the ALAS1+/− model suggests that accelerated heme loss accelerates aging, and conversely, maintaining heme levels could slow certain age-associated pathologies. This provides a strong rationale for a deeper look into heme-targeted geroprotective therapies, especially given that heme, particularly free heme, has traditionally been viewed negatively in the context of health [45]. These aging-related phenotypes underscore the systemic importance of maintaining heme homeostasis. However, organ-specific susceptibilities further clarify the diverse consequences of heme deficiency, as discussed next.

## 7. Multiorgan Impact of Heme Deficiency

Having detailed our group’s core findings on systemic metabolism, mitochondrial dysfunction, and accelerated aging in ALAS1+/− mice, we now extend our perspective to examine organ-specific consequences that remain less fully explored but provide possible avenues for future research. Beyond metabolic and muscular phenotypes, heme deficiency causes dysfunction in multiple organ systems. While previous sections focused on global metabolic and muscular impacts, heme deficiency also disrupts key organ specific processes. The following sections outline its effects on erythropoiesis, innate immunity, and neuronal function, highlighting the widespread biological dependence of the body on adequate heme availability.

### 7.1. Disrupted Erythropoiesis and Anemia in ALAS2 Deficiency

Adequate heme supply is indispensable for red blood cell production. In developing erythroid cells, heme is required for hemoglobin synthesis. Without it, erythropoiesis is impaired, leading to anemia. In animal models, even partial disruption of heme synthesis leads to anemia [46]. ALAS2+/− female mice, which carry a heterozygous loss-of-function allele on the X chromosome, exhibit microcytic hypochromic anemia due to a reduced hemoglobinization of erythrocytes. Complete loss of ALAS2 is embryonically lethal due to severe anemia, as discussed earlier in Section 2. In less severe cases, heme deficiency leads to sideroblastic anemia, where iron is imported into developing red cells but cannot be incorporated into heme. As a result, it accumulates around the mitochondria, forming ring sideroblasts. As noted in Section 2, XLSA is a clinical example of disrupted heme biosynthesis, and is associated with chronic anemia and systemic iron overload due to compensatory intestinal iron absorption [47]. Similarly, mice with mutations in downstream heme pathway enzymes show anemia and elevated free iron, reflecting ineffective erythropoiesis [48]. Clinically, anemia from heme or iron deficiency manifests as fatigue, pallor, weakness, and sometimes tachycardia [49,50]. Standard treatments include pyridoxine (vitamin B6), especially when ALAS2 mutations impair cofactor binding, or transfusions in severe cases [51]. In some instances, heme arginate or 5-ALA can bypass biosynthetic blocks to restore heme levels [52,53].

### 7.2. Stress Signaling Through the HRI-ISR Axis in Anemic States

At the cellular level, heme deficiency activates HRI, especially in reticulocytes. HRI plays a protective role by halting globin synthesis when heme is unavailable, thus preventing the accumulation of unpaired, misfolded globin chains [54]. However, sustained activation of HRI induces the ISR, leading to ATF4-driven transcription of stress genes. If stress persists, erythroid precursors undergo apoptosis before maturing [55].

The ISR is a convergence point for multiple stressors: heme deficiency, oxidative stress, ER stress, and nutrient deprivation. Four kinases, PERK, GCN2, PKR, and HRI, phosphorylate eIF2α to initiate translational control and stress resolution. During heme-deficient erythropoiesis, HRI-driven ISR may overlap with other stress pathways, including the unfolded protein response (UPR) initiated by ER-localized PERK [56].

In summary, (sideroblastic) anemia is the most direct and well-established consequence of heme deficiency, rooted in a failure of hemoglobin synthesis and coupled with maladaptive stress signaling that limits red blood cell survival. The extent to which ALAS2 function overlaps with that of ALAS1 remains to be elucidated. Interestingly, a study by Peng et al. reported that overexpression of ALAS2 led to muscle atrophy and mitochondrial dysfunction [57], raising the possibility that ALAS2 may also influence skeletal muscle health and metabolism under certain conditions, highlighting the need to further explore its potential roles beyond erythropoiesis.

## 8. Innate Immune System Suppression Under Heme-Deficient States and Restoration by 5-ALA

Heme plays an often-underappreciated role in the immune system. Immune cells utilize heme-containing enzymes for their function; for example, NADPH oxidase in neutrophils, which generates reactive oxygen to kill bacteria, has heme prosthetic groups [58]. Heme is also needed for the enzyme nitric oxide synthase (NOS), which produces nitric oxide by neutrophils [59], and heme also has an important function for guiding inflammation, either pro- or anti-inflammatory, for macrophages [60]. A study by Saitoh et al. 2024 using our ALAS1+/− mice revealed that heme deficiency impairs innate immune responses [61].

When challenged with endotoxic shock bylipopolysaccharide (LPS) injection, ALAS1+/− mice show reduced mortality compared to wild-type, but this apparent protection is due to an inadequate immune activation. Heme-deficient mice failed to mount the normal inflammatory cytokine surge as tumor necrosis factor-α (TNF-α) and interleukin-6 (IL-6) were significantly lower than in the wild type. Interestingly, pharmacologic inhibition of heme synthesis by SA similarly blunts LPS-induced TNF α and IL-6 release, suggesting that disruption of heme availability genetically or pharmacologically impairs cytokine production [62].

ALAS1+/− mice also exhibited reduced recruitment of immune cells to sites of infection. For instance, fewer neutrophils migrated into the peritoneal cavity during peritonitis in ALAS1+/− mice. This mirrors observations in models of HO-1 overexpression: excessive heme catabolism via HO-1 activation suppresses neutrophil infiltration and dampens TNFα cytokine release following LPS challenge [63,64]. Together, these findings suggest that balanced heme metabolism is critical for mounting a robust innate immune response [61].

Ex vivo experiments confirmed that the immune defect is directly related to heme/5-ALA availability. Bone marrow-derived macrophages and dendritic cells (from wild-type mice) showed enhanced activation of inflammatory genes when pre-treated with 5-ALA (and ferric iron), indicating that improving heme synthesis heightens their responsiveness [61]. Corroborating on the above, ex vivo heme synthesis dependence studies demonstrate that increase in heme levels in macrophages increases ROS generation, NO production, and cytokine/IFN-β signaling, thus improving phagocytosis and bacterial killing [65].

Neutrophils from ALAS1+/− mice were found to have deficient bactericidal activity; their phagocytosis and ROS generation were notably lower than wild-type neutrophils. After two weeks of oral 5-ALA administration to ALAS1+/− mice, neutrophil ROS production and phagocytic ability were largely restored. These results show that heme is required for the ROS-generating response of neutrophils following activation [61]. This is also supported by zebrafish *alas1*-deficient models demonstrating impaired neutrophil maturation, absent granule proteins, and reduced bacterial clearance [66].

The research above suggests that heme-deficient animals are more susceptible to infections. They are more likely to fail at efficiently clearing bacteria due to impaired recruitment and reduced function of neutrophils and macrophages. Also, their reduced cytokine response can hamper the coordination of immune defense. Notably, HO-1 is often upregulated during inflammation as a feedback modulator [67]; however, in a state of heme deficiency, HO-1 induction might be blunted [18], potentially affecting iron recycling and resolution of inflammation. Upon LPS stimulation, 5-ALA and ferric iron-supplemented wild-type mice did not have an increase in HO-1 expression, although it is worth noting that treatment of extracted bone marrow-derived cells only lasted for one day. If treatment is prolonged, 5-ALA through HO-1 induction could possibly enhance macrophage and neutrophil activity [68] and attain anti-oxidant and immunomodulatory effects [15,69]. Meanwhile, 5-ALA in wild-type mice did increase phagocytosis capability and increased *Il6* and *Tnfa* mRNA expression. These findings support the potential of 5-ALA supplementation to restore cytokine production and microbicidal function, highlighting nutritional heme augmentation as a strategy to improve innate immunity in immunocompromised patients.

## 9. Emerging Roles of Heme in Neuronal Health

Although the primary focus of our review centers on muscle and metabolic dysfunction in heme deficiency, and while the central nervous system has not been comprehensively studied in our mouse models, emerging evidence points to an equally important role of heme in maintaining neuronal health, with heme being crucial for neuronal metabolism, mitochondrial maintenance, neurotransmission, and redox homeostasis. Below, we summarize emerging evidence linking heme homeostasis to brain health, including recent data supporting 5-ALA as a candidate therapeutic for neurodegenerative disorders. Heme supports several key neuronal processes, including neurotransmitter synthesis, via tyrosine and tryptophan hydroxylases, N-methyl-D-aspartate (NMDA) receptor activity, nitric oxide signaling, and mitochondrial electron transport [70,71,72,73].

In addition to neurons, oligodendrocytes express the heme transporter Hrg1, which is essential for maintaining myelin integrity [74].

In vitro studies show that blocking heme synthesis in neural cells leads to apoptosis and impaired neurite outgrowth. For example, treatment with SA suppresses expression of neuroprotective genes and activates stress responses such as Hsp70 and Hsp27, while heme supplementation rescues ERK1/2 signaling and cell viability [75,76]. Likewise, heme deprivation impairs NMDA receptor function, which can be restored with heme [76], suggesting that neuronal redox and plasticity pathways require adequate amounts of heme.

Recent findings also point to potential therapeutic applications of heme precursors in neurodegeneration. A study by Matsuo et al., 2024, showed that oral 5-ALA treatment prevented α-synuclein aggregation and halted motor decline in a Parkinson’s disease (PD) mouse model [77]. Other reports suggest HO-1 induction by 5-ALA also mitigates oxidative stress in PD rats [78]. These results align with our observations of 5-ALA improving mitochondrial function and possibly reducing oxidative stress in other tissues.

In Alzheimer’s disease (AD), several studies have reported features consistent with cerebral heme deficiency. Postmortem analyses of AD brains show reduced cytochrome c oxidase activity, iron accumulation, and lower heme levels in affected neurons [70]. Some researchers have proposed that impaired heme biosynthesis, possibly due to downregulation of ALAS1 or insufficient vitamin B6, may contribute to mitochondrial dysfunction and enhance amyloid pathology. While the exact role of heme remains debated, heme has been shown to bind to amyloid-β peptide, potentially influencing its aggregation and redox activity [79,80].

While excess free heme is neurotoxic, for example, during hemorrhagic stroke, controlled heme restoration may support central nervous system function, especially in conditions marked by mitochondrial dysfunction or oxidative stress. Given the brain’s high autophagy rate and redox sensitivity, age-related heme decline may subtly contribute to neurodegenerative risk, highlighting the need to further investigate the role of heme homeostasis in neuronal health and its therapeutic potential [30]. Together with the recent studies in PD, these findings suggest that boosting heme synthesis or bioavailability could represent a novel therapeutic angle in neurodegenerative diseases marked by mitochondrial compromise.

## 10. Conclusions and Future Perspectives: Heme as a Multisystem Regulator and Therapeutic Target

**Figure 1:**
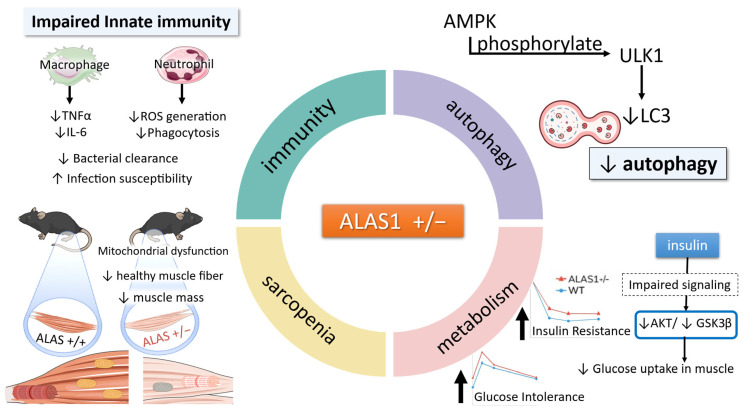
Summary of systemic pathologies observed in ALAS1+/− mice. Focus lies on the metabolic, muscular, immune, and sarcopenia-related phenotypes identified in our studies of ALAS1-heterozygous mice.

Heme is an indispensable molecule whose deficiency leads to multi-system pathology. Mouse models and cell studies show that even moderate heme synthesis reduction, as in ALAS1+/− mice, can cause features of metabolic syndrome (insulin resistance, glucose intolerance), myopathies (mitochondrial dysfunction, sarcopenia), immune deficiencies (blunted cytokine responses, reduced microbial killing), and accelerated aging phenotypes (Figure 1). More severe heme deficits result in anemia and embryonic lethality. These findings emphasize that heme is not merely a static cofactor but a dynamic regulator of cellular stress responses and systemic adaptation.

Mechanistically, heme deficiency disrupts essential biological processes: it impairs mitochondrial oxidative phosphorylation, causes ineffective erythropoiesis and iron mismanagement, activates the UPR-ISR via eIF2α kinases like HRI [54], alters gene expression through sensors like Bach1 and Reverbα, and suppresses key metabolic regulators such as AMPK. The downstream effects—energy failure, ROS accumulation, and chronic stress—promote apoptosis, senescence, and tissue dysfunction.

These pathological consequences can be reversed or mitigated by restoring heme levels. In ALAS1+/− mice, 5-ALA administration normalized glucose tolerance, improved mitochondrial function and exercise capacity, restored autophagy markers, and enhanced immune responsiveness. These preclinical results support the potential of 5-ALA as a therapeutic agent for heme-deficient conditions.

Recent interest in intracellular heme trafficking has also uncovered proteins such as GAPDH and PGRMC2 as critical regulators of heme delivery and signaling [24,81]. Modulating these chaperones may offer alternative strategies to influence metabolism and stress resistance without broadly altering total heme levels. Notably, PGRMC2 has been shown to preserve metabolic health in obese mouse models, highlighting its therapeutic relevance beyond classical heme biosynthesis.

In summary, heme is a central nexus in mitochondrial function and cell physiology. Its deficiency links a range of disorders—anemia, diabetes, muscle wasting, neurodegeneration, and immune dysfunction—through shared impairment of heme-dependent processes. These pathologies are summarized in Table 1, together with therapeutic implications discussed in this review.

Our group’s pioneering work with ALAS knockdown models has elucidated many of these connections, demonstrating that heme homeostasis is critical throughout life. These insights open the door to novel therapeutic strategies, including the use of 5-ALA supplementation and pharmacological targeting of heme-responsive sensors.

While these findings provide insight into the role of heme availability in age-related metabolic dysfunction, important questions remain regarding their translational relevance. Rodent models may not fully capture the complexity of human aging or heme regulation, and the dose and bioavailability of 5-ALA may vary across species. Nevertheless, 5-ALA is already approved for human use (e.g., in photodynamic diagnosis) and has been evaluated in clinical trials for metabolic disorders such as mitochondrial diabetes [82,83]. Future studies should determine whether age-related declines in heme levels occur in humans, and whether 5-ALA supplementation improves quality of life in older adults. Controlled trials and biomarker-driven studies will be essential to validate its therapeutic potential.

In an aging society, 5-ALA supplementation could conceivably be repurposed to improve lifespan and health span by addressing an often-overlooked but tractable contributor: the age-related decline in heme availability.

## Figures and Tables

**Table 1:** Overview of ALAS1/ALAS2-linked pathologies and therapeutic benefits of 5-ALA treatment across studies.

Related Gene/Compound	Pathology or Benefit	Article or Study Reference
ALAS1	Embryonic lethality (E7.5), developmental arrest	Okano et al., 2010 [16]
ALAS1	Impaired glucose tolerance, insulin resistance, mitochondrial dysfunction	Saitoh et al., 2018 [17]
ALAS1	Reduced autophagy, sarcopenia (AMPK supression)	Akabane et al., 2024 [23]
ALAS1	Suppressed immune response (impaired cytokine production, impaired neutrophil function)	Saitoh et al., 2024; [61]
ALAS1	Glycogen storage (AMPK supression)	Nakajima et al., 2018 [27]
ALAS2	Embryonic lethality (~E11.5), severe anemia, impaired erythropoiesis, iron overload	Nakajima et al., 1999 [10] Ono et al., 2022 [21]
ALAS2	X-linked sideroblastic anemia (XLSA), mitochondrial iron accumulation in erythroblasts	Aivado et al., 2006 [19] Nakajima et al., 2006 [20]
ALAS2	Muscle atrophy, mitochondrial dysfunction (overexpression study)	Peng et al., 2018 [57]
5-ALA	Improved glucose tolerance, insulin sensitivity, mitochondrial function, improved muscle mass and muscle function	Fujii et al., 2017 [26]
5-ALA	Enhanced immune response, restored cytokine production, neutrophil function, and bacterial clearance	Saitoh et al., 2024 [61]
5-ALA	Reduced α-synuclein aggregation and motor decline (Parkinson’s disease model)	Matsuo et al., 2024 [77]

## Data Availability

Not applicable.

## List of Abbreviations

5-ALA	5-aminolevulinic acid
AD	Alzheimer’s disease
ALAS	ALA synthase
ALAS1+/−	ALAS1 heterozygous
ALAS1–/–	Alas1 knockout
ALAS2+/−	ALAS2 heterozygous
AMPK	AMP-activated protein kinase
DEPC5	DEP domain-containing 5
ETC	electron transport chain
GYS1	glycogen synthase
HO-1	heme oxygenase-1
HRI	heme-regulated inhibitor kinase
IL-6	interleukin-6
ISR	integrated stress response
LPS	lipopolysaccharide
MHO-1	skeletal muscle-specific HO-1 deletion
mTORC1	mammalian target of rapamycin complex 1
NMDA	N-methyl-D-aspartate
NOS	nitric oxide synthase
PD	Parkinson’s disease
PGRMC2	progesterone receptor membrane component 2
ROS	reactive oxygen species
SA	succinyl acetone
TNF-α	tumor necrosis factor-α
UPR	unfolded protein response
XLSA	X-linked sideroblastic anemia
